# Supplementary material for: Community metabolic modeling of host-microbiota interactions through multi-objective optimization
Source: iScience. 2024 May 23;27(6):110092. doi: 10.1016/j.isci.2024.110092 (PMC11215293; doi:10.1016/j.isci.2024.110092)
Supplement: Document S1. Figures S1‒S5 and Tables S2 and S3 [file mmc1.pdf]

iScience, Volume *27*

Supplemental information

Community metabolic modeling of host-microbiota interactions through multi-objective optimization

Anna Lambert, Marko Budinich, Maxime Mahé, Samuel Chaffron, and Damien Eveillard

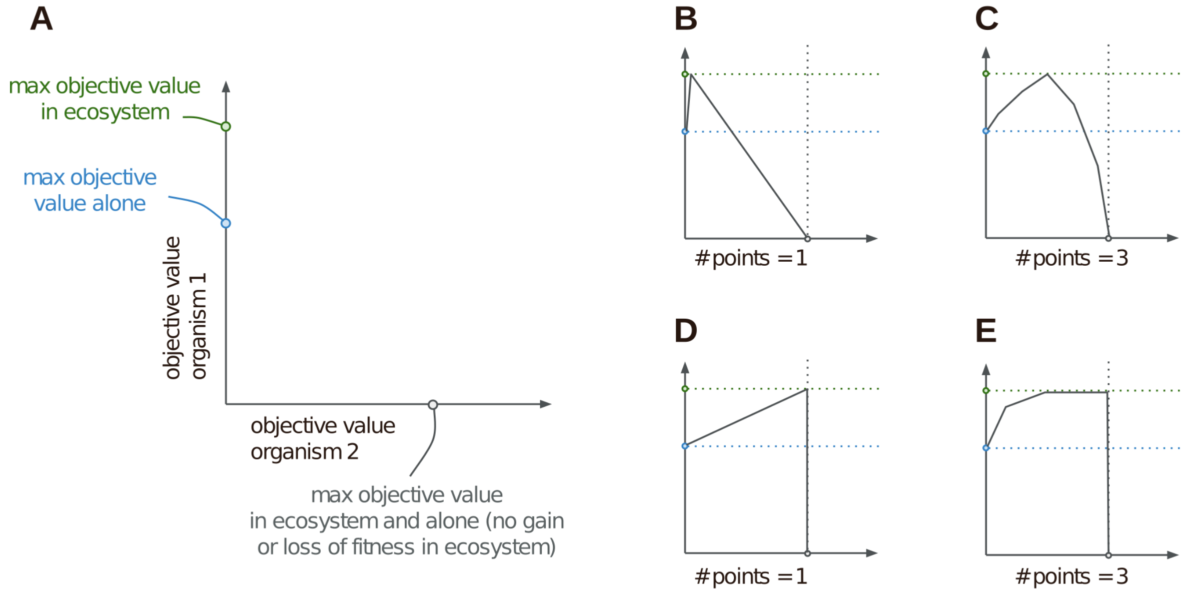

Figure. S1 | Illustration of the improved characterization of the ecosystem interaction through Pareto front inference rather than comparison of growth and co-growth rates, related to Figure 1. (A) Information extracted from a method comparing growth (i.e. alone) and co-growth (i.e. in an ecosystem) rates of an organism[1]. In this scenario, organism 1 achieves a higher objective value in the presence of organism 2 compared to when alone, while the maximal objective value of organism 2 remains constant in both conditions. Such methods can conclude that organism 1 grows faster in co-growth simulation. This situation can be explained by multiple Pareto front, with a selection represented in **(B)**, **(C)**, **(D)**, and **(E)**. The different Pareto fronts translate into various interaction dynamics, illustrating the added value of computing these trade-offs. Inferring the interaction score of these Pareto fronts automates the discrimination of these interaction dynamics.

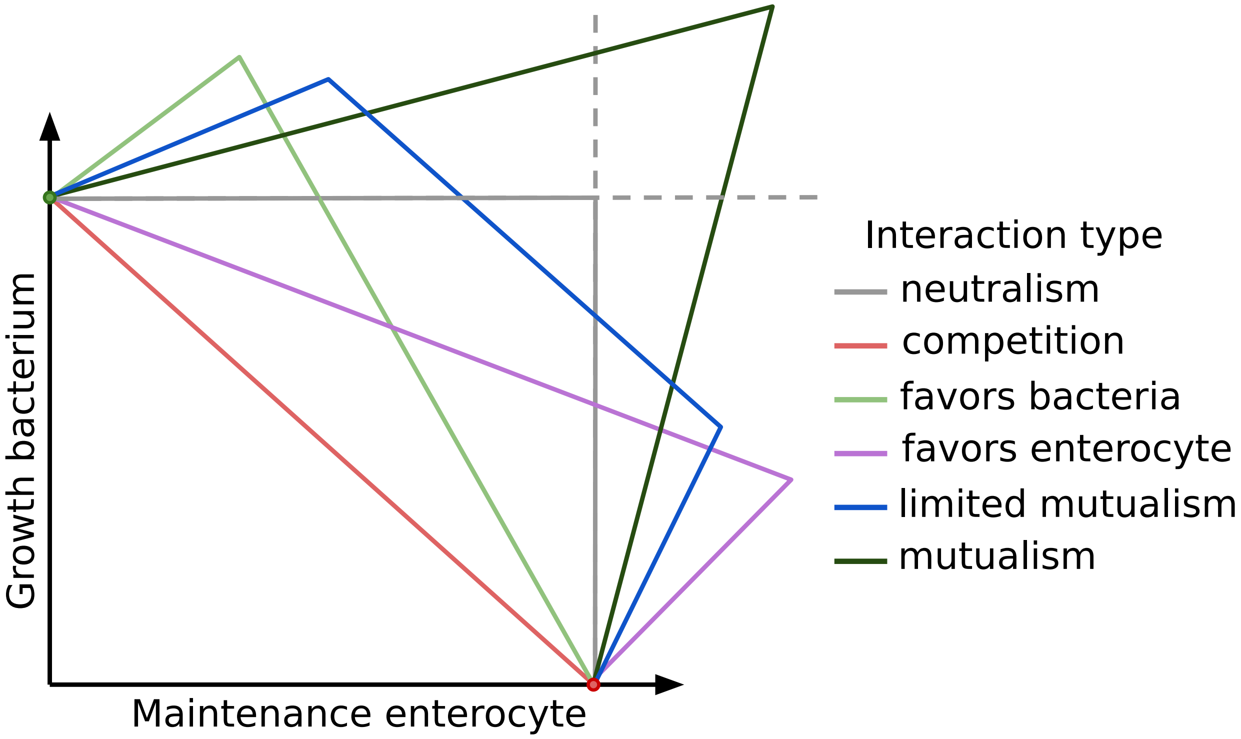

Figure. S2 | Illustration of the interaction types inferred from the Pareto shapes, related to STAR Methods.

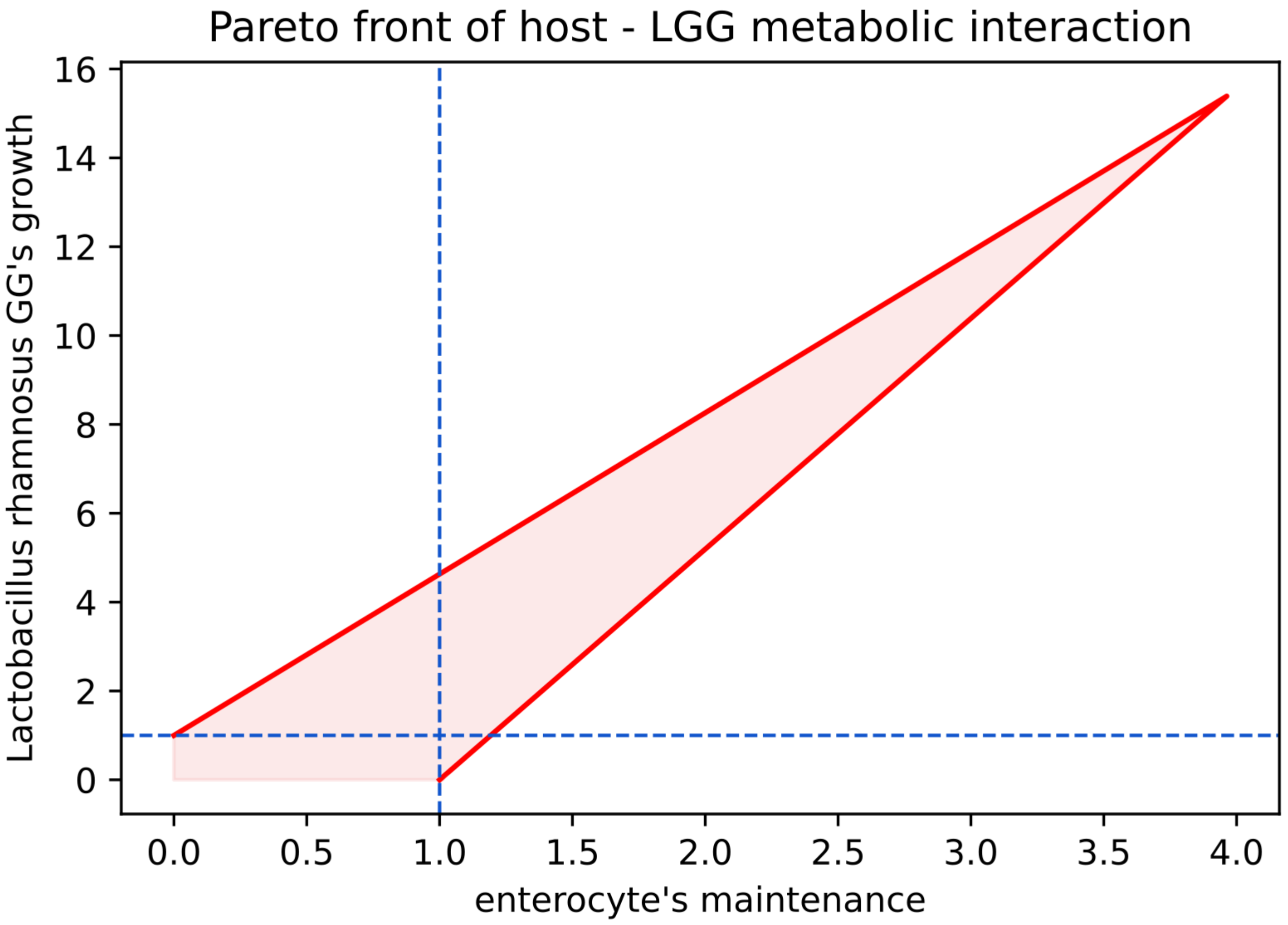

Figure. S3 | Pareto front of the interaction between the enterocyte and Lactobacillus rhamnosus GG in a Western diet, related to Figure 3.

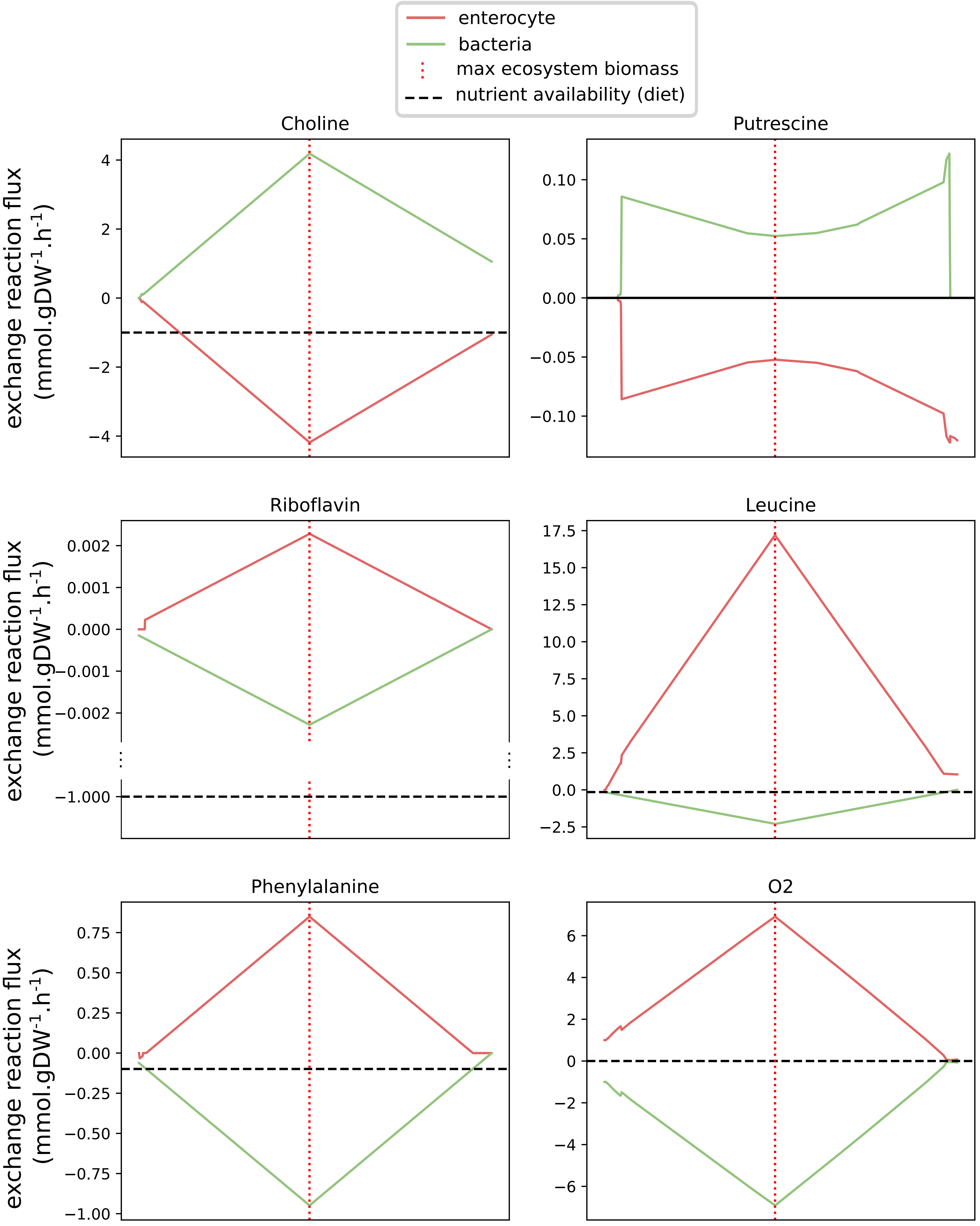

Figure. S4 | Exchanged metabolites between the enterocyte and Lactobacillus rhamnosus GG in a Western diet. Colored lines display the flux going through the transport reaction of a given metabolite, evolving along the Pareto front, related to Figure 3.

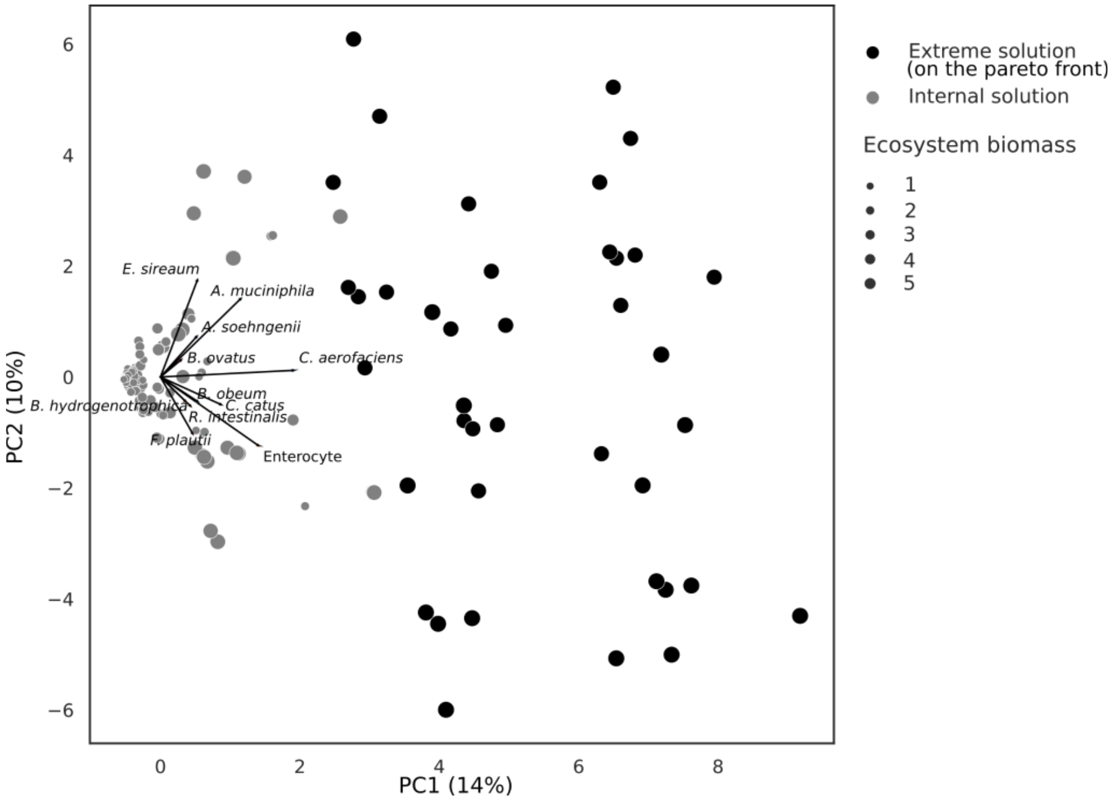

Figure. S5 | PCA on the objective space of the multi-objective community metabolic modeling of 11 organisms (enterocyte, *A. muciniphila, A. soehngenii, B. hydrogenotrophica, B. obeum, B. vatus, C. aerofaciens, C. catus, E. sireaum, F. plautii, R. intestinalis*) in an ecosystem. Computational resources enabled the multi-objective analysis of 10 gut bacteria joined with the enterocyte. Among the 16 strains representing a minimal gut microbiome according to Shetty et. al.[1], we selected the ten strains from with the smallest niche overlap (*Akkermansia muciniphila* MucT/ATCC BAA-835, *Anaerobutyricum soehngenii* L2-7/DSM 17630, *Blautia hydrogenotrophica* DSM 10507*, Blautia obeum* DSM 25238*, Bacteroides ovatus* HMP strain 3_8_47FAA*, Collinsella aerofaciens* DSM 3979*, Coprococcus catus* ATCC 27761*, Eubacterium sireaum* DSM 15702*, Flavonifractor plautii* HMP strain 7_1_58FAA*, Roseburia intestinalis* DSM 14610).

To explore how organisms influenced each other's objectives, we realized a PCA on both the extreme points of the Pareto front and 3000 random solutions sampled in the objective space (Figure S5). Similarly to the results observed in the five-dimensional ecosystem described in the core paper, PC1 is overall associated with the ecosystem biomass value. Here again, each organism participates in the increase of biomass production in the ecosystem. However, the variance explained by PC1 and PC2 only reaches 24% and the high-dimensional nature of the data makes it difficult to raise many conclusions from the directions taken from each organism on the PCA. Additionally, every solution on the Pareto front implied the absence of growth for at least one organism of the ecosystem, implying total competition between some organisms. Finally, the interaction score was calculated for a reduced ecosystem, removing one organism each time, to evaluate the impact of each organism on the overall positive or negative interaction of the ecosystem. This resulted in only scores of -1, hinting towards a highly competitive ecosystem.

Table. S2 | Growth of mutualist bacteria and the host, in isolation or in ecosystem, in WD or PD, related to Figure 2.
An increased score is not necessarily due to an improved growth, but rather to a higher interdependance among organisms.

diet		host	Lactobacillus paracasei ATCC 334	host	Peptoniphilus indolicus ATCC 29427	host	Lactobacillus rhamnosus GG	host	Bifidobacterium scardovii JCM 12489 = DSM 13734	host	Corynebacterium kroppenstedtii DSM 44385	host	Barnesiella intestinihominis YIT 11860
WD	Bini_WD	0,064	0,344	0,064	0,344	0,064	0,344	0,064	0,368	0,064	0,283	0,064	0,403
	Beco_WD	0,253	5,290	0,065	5,290	0,253	5,290	0,065	5,290	0,620	5,290	0,253	5,290
PD	Bini_PD	0,068	0,688	0,068	0,688	0,068	0,688	0,068	0,736	0,068	0,566	0,068	0,806
	Beco_PD	0,507	5,290	0,069	5,290	0,507	5,290	0,069	5,290	1,240	5,290	0,507	5,290

Bini : Maximal biomass of the organism when alone
Beco : Maximal biomass of the organism in the ecosystem

Table. S3 | Minimal microbiome genome references, related to Figure 4

specie	strain	ncbi_id	Accession number	quality
Akkermansia muciniphila	ATCC BAA-835	349741	GenBank :GCA_000020225.1	Complete genome
Bacteroides xylanisolvens	HMP strain 2_1_22	38349	GenBank :GCA_000162155.1	"scaffold" (High_quality draft)
Faecalibacterium prausnitzii	A2-165	853	GenBank :GCA_002734145.1	Complete Genome
Ruminococcus bromii	ATCC 27255	40518	GenBank :GCA_002834225.1	contig
Coprococcus catus	ATCC 27761	116085	GenBank :GCA_025289135.1	Scaffold
Flavonifractor plautii	HMP strain 7_1_58FAA	39397	GenBank :GCA_000242155.1	"scaffold" (High_quality draft)
Eubacterium sireaum	DSM 15702	428128	GenBank :GCA_000382085.1	scaffold
Agathobacter rectalis	DSM 17629	657318	GenBank :GCA_000209935.1	Chromosome
Roseburia intestinalis	DSM 14610 (L1-82)	166486	GenBank :GCA_900537995.1	Complete Genome
Anaerobutyricum soehngenii	L2-7/DSM 17630	105843	GenBank :GCA_009697165.1	Contig
Subdoligranulum variabile	DSM 15176	411471	GenBank :GCA_000157955.1	Scaffold
Blautia obeum	DSM 25238 (= ATCC 29174)	40520	GenBank :GCA_025147765	complete
Collinsella aerofaciens	DSM 3979 (=ATCC 25986)	74426	GenBank :GCA_000169035	contig
Bifidoabcterium adolescentis	L2-32	411481	GenBank :GCA_000154085.1	Scaffold
Blautia hydrogenotrophica	DSM 10507	476272	GenBank :GCA_000157975	scaffold
Bacteroides ovatus	HMP strain 3_8_47FAA	40011	GenBank :GCA_000218325.1	"scaffold" (High_quality draft)

reference genome, not strain (strain not found)

1. Shetty, S.A., Kostopoulos, I., Geerlings, S.Y., Smidt, H., de Vos, W.M., and Belzer, C. (2022). Dynamic metabolic interactions and trophic roles of human gut microbes identified using a minimal microbiome exhibiting ecological properties. ISME J. *16*, 2144–2159. 10.1038/s41396-022-01255-2.
